# Advanced gynecological cancer: Quality of life one year after diagnosis

**DOI:** 10.1371/journal.pone.0287562

**Published:** 2023-06-23

**Authors:** Björg Jónsdóttir, Anna Wikman, Inger Sundström Poromaa, Karin Stålberg

**Affiliations:** Department of Women’s and Children’s Health, Uppsala University, Uppsala, Sweden; Sapienza University of Rome: Universita degli Studi di Roma La Sapienza, ITALY

## Abstract

**Objective:**

Gynaecological cancer treatment impacts women’s physical and psychological health. Our objective was to examine quality of life (QoL) in women with advanced gynaecological cancer at diagnosis and one year later, and to identify sociodemographic and clinical characteristics associated with QoL.

**Methods:**

Women with endometrial, ovarian or cervical cancer treated in Uppsala, Sweden 2012–2019 were included. FIGO stage ≥II was considered advanced gynaecological cancer, whereas women in FIGO stage I were used as a control group. QoL was assessed with SF-36. We obtained information on sociodemographic and clinical characteristics from medical records and health questionnaires. Differences in QoL domains were tested with t-tests, a mixed model ANOVA and multiple linear regression analyses.

**Results:**

The study population (n = 372) included 150 (40.3%) women with advanced gynaecological cancer. At diagnosis, women with advanced cancer reported lower physical (71.6 vs 81.8 (mean) p<0.05) and role functioning/physical scores (62.6 vs 77.2 (mean) p<0.05) than women in FIGO stage I. One year later, women with advanced cancer reported higher scores in the mental health domain (78.3 vs 73.2 (mean) p<0.05) than women in FIGO stage I. However, no difference was found in the QoL scores of women with advanced disease one year after diagnoses when stratified by diagnosis. Women with a history of psychiatric illness and higher BMI reported poorer physical and mental QoL at follow-up, while advanced stage, level of education and smoking were not associated with QoL.

**Conclusion:**

Women with advanced gynaecological cancer have equally good QoL one year after diagnosis as women with limited disease. Women with previous psychiatric illness and high BMI, are at risk of impaired physical and mental health.

## Introduction

Every year, around 2200 women are diagnosed with endometrial, ovarian and cervical cancer in Sweden [1]. In advanced cancer, FIGO stage ≥II, the treatment is often a combination of extensive surgery and chemotherapy and/or radiotherapy. The diagnosis and treatment impact on these women’s quality of life (QoL), leaving them at risk of anxiety and depression and impairing their physical and emotional functioning. Common problems in cancer survivors are pain, fatigue, anxiety, distress and depression [2]. For most women, these problems improve over the first year after diagnosis, while others report ongoing physical and mental health problems [3]. It is desirable to identify individuals at risk of prolonged physical and mental health problems, in order to offer the right support.

Previous studies indicate that the severity of surgery and pretreatment performance status are predictive factors of poor QoL [4] along with disengaged coping at diagnosis (which is also associated with greater stress [3]). Other factors associated with poor QoL are physical comorbidities, lower socioeconomic status and living alone [5, 6]. Younger age has been associated with poorer emotional functioning while older patients show poorer physical functioning [7–10]. Age can therefore influence quality of life in many ways and frailty of older patients can further influence their oncological outcome [11]. In addition, advanced tumor stage, radiotherapy and chemotherapy have been associated with poorer QoL [12].

In one meta-analysis the prevalence of depression in gynecological cancer patients was 23%, which was higher than for most other cancer diagnoses [13]. Women, in general, have higher rates of anxiety and depression, and after cancer diagnosis more than 50% of women reach subclinical or clinical levels of anxiety [14]. Anxiety usually improves with time and treatment but persisting depression and diminished QoL have been associated with pain medication use at baseline and to holding back concerns [15].

In ovarian cancer the level of anxiety symptoms is higher than the corresponding level of depressive symptoms and is highest prior to surgery, gradually decreasing thereafter. In this cancer group the main determinants of distress are the presence of intestinal stoma, poor general health status and residual disease [16]. However the overall QoL for ovarian cancer survivors is generally good compared to healthy women, despite the persistence of psychological and physical symptoms [17]. Although many adjust well after treatment, others, including women with multiple recurrent disease, appear to have a more impaired QoL [18].

In endometrial cancer, obesity is associated with poorer QoL and physical functioning. Furthermore, treatment with laparoscopy (vs laparotomy) and vaginal brachytherapy (vs external beam radiation) is favorable. Sexual function outcomes seem dependent on age and time since diagnosis [19]. In cervical cancer survivors, better QoL is found in women with a current occupation, a longer time since treatment, and among those who have undergone hysterectomy (vs chemo and radiotherapy) [20]. In long-term survivors of both cervical and endometrial cancer, cervical cancer patients report more anxiety, dysphoria, anger and confusion, while both groups report depression to a similar extent [5].

The treatment of women with gynecological cancers is decided mainly by their FIGO (International Federation of Gynecology and Obstetrics) stage, where women in FIGO stage I are usually only treated with surgery while women in stage ≥II (i.e., advanced cancer) are most often treated with chemotherapy and/or radiotherapy with or without surgery. Previous studies often include women in all FIGO stages, and information is lacking on the differences in QoL of women with advanced disease (and treatment) compared to women with localized disease. Thus, the aim of this study was to examine QoL in women with advanced gynecological cancer at diagnosis and one year later, compared with women with earlier tumor stage. Furthermore, we aimed to identify the sociodemographic and clinical characteristics of each of the eight QoL domains at the one-year follow-up. Due to worse prognosis in combination with extended treatment we hypothesized that women with advanced cancer would have poorer QoL one year after diagnosis than women in stage I.

## Material and methods

### Study design and data source

This was a cross-sectional analysis of data obtained from the Uppsala-Umeå Comprehensive Cancer Consortium (U-CAN), Sweden. The U-CAN study was initiated in 2010 by Uppsala and Umeå Universities and their corresponding university hospitals. The aims of U-CAN are to gather a high-quality longitudinal biobank and a database of clinical information on a large number of patients to facilitate translational and clinical research [21]. U-CAN is thus a systematic prospective longitudinal sampling of multidisciplinary clinical data, together with blood and tissue samples from cancer patients. Gynecological cancers were included in U-CAN from January 2012 and onwards, only with reference to patients from Uppsala University Hospital [21].

Participating in U-CAN includes giving blood samples, providing fresh frozen or formalin-fixed paraffin embedded tissue specimens, undergoing radiology and answering questionnaires about different aspects of health and well-being. All patients included in the U-CAN database gave written informed consent.

The study procedures were in accordance with ethical standards for human experimentation and the study was approved by the local ethics committee (DNR 2010–19811, 2020–06824).

### Study population

**Fig 1:**
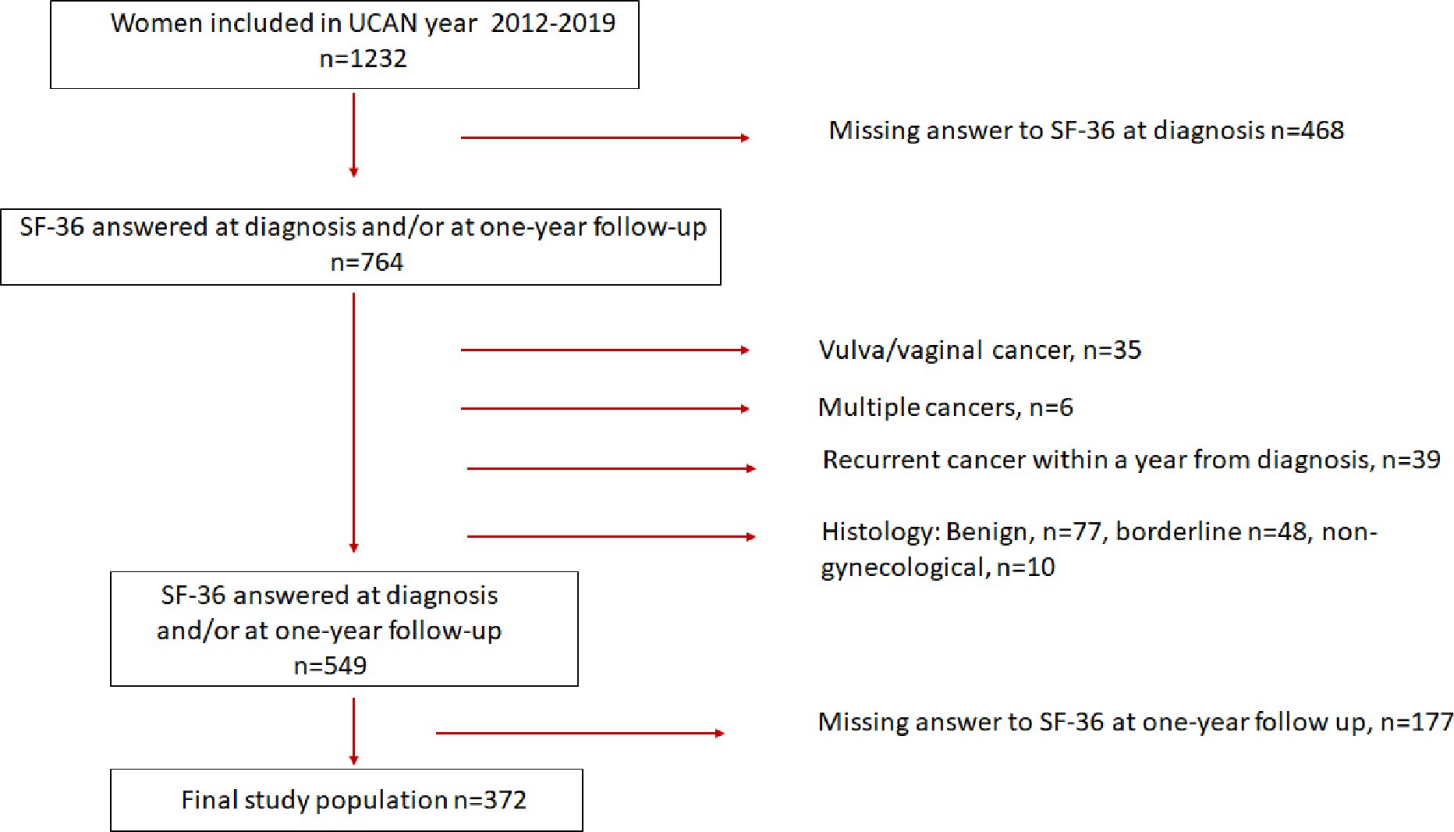
Flow chart defining the study population.

A total of 1232 women treated at the Uppsala University Hospital for endometrial, ovarian or cervical cancer during 2012–2019 were eligible for inclusion in this study. The inclusion criterion for the present study was completing the Short Form 36 Health Survey, version 2 (SF-36) at diagnosis and at the one-year follow-up. Exclusion criteria were another histology, multiple cancers, recurrent cancer and missing answers on the SF-36 at the one-year follow-up. The final study population consisted of 372 women (Fig 1) where the most common cause for exclusion was women not answering the SF-36 (n = 468).

### Sociodemographic and clinical characteristics

Information on sociodemographic and clinical characteristics was obtained from the U-CAN general health questionnaire and completed with data from medical records. Information from medical records included cancer type (ovarian, endometrial or cervical cancer), number of treatment modalities (1, 2 or 3), age, body mass index (BMI, kg/m^2^), American Society of Anesthesiologists (ASA) score [22] (10, 20, 30 or 40) and FIGO stage (I or ≥II). Women in FIGO stage ≥II were classified as having advanced gynecological cancer.

The following information was included from the U-CAN general health questionnaire: number of comorbidities (including diabetes mellitus, hypertension, hypothyroidism, liver disease, cardiac infarction, angina pectoris, cardiac failure, cerebral stroke/ischemia, high cholesterol, lung disease, inflammatory bowel disease, kidney disease, previous cancer diagnosis), smoking (yes or no), level of education (university or not university level), living situation (with partner or living alone), alcohol consumption (units per week) and history of psychiatric illness (yes or no). The information on psychiatric illness was further confirmed with data from medical records.

### Quality of life

Quality of life at diagnosis and the one-year follow-up was assessed using the SF-36v, a multi-purpose, short form health survey containing 36 questions across eight domains. The eight domains of the SF-36v are designed to capture the most frequently measured issues in health surveys and those that are most affected by disease and treatment [23], and include both physical (physical functioning, role functioning/physical, bodily pain, general health) and mental (vitality, social functioning, role functioning/emotional, mental health) health. This questionnaire has been widely used since its publication in June 1992 and more than 2000 articles including SF-36 have been published [24]. In Sweden the questionnaire was translated and tested in the Swedish population in the years 1990–1991. Consequently, normative values for the Swedish population are available, based on a study of 8930 individuals from seven different areas in the country [25, 26].

### Statistical analyses

Attrition analyses were carried out to examine differences in sociodemographic and clinical characteristics between women who were included in the study (i.e., women answering the SF-36 at both diagnosis and at the one-year follow-up) and non-responders (i.e., women answering the SF-36 only at diagnosis), using an independent-samples t-test. Data were presented using descriptive statistics describing means and standard deviations (sd) (or median with inter-quartile range [IQR], for non-normally distributed variables) for numerical variables and numbers (n) and percentages (%) for categorical variables. Scores on each of the eight QoL domains at diagnosis and at the one-year follow-up between women with advanced cancer (i.e., FIGO stage ≥II) or not (i.e., FIGO stage I) were compared using an independent-samples t-test. Changes in QoL scores over time were analyzed with a mixed model ANOVA. The SF 36 scores of women with FIGO stage ≥II were further grouped according to gynecologic cancer diagnosis (endometrial and ovarian cancer) and compared with independent-samples t-tests. Women with cervical cancer were not included due to low number of cases with advanced disease. Sociodemographic and clinical characteristics in relation to each of the eight QoL domains at the one-year follow-up were analyzed using multiple linear regression. The results are presented as unstandardized B coefficients with 95% confidence intervals. All analyses were carried out using IBM SPSS statistics 24 (IBM Corp. Released 2016. IBM SPSS Statistics for Windows, Version 24.0. Armonk, NY: IBM Corp).

## Results

### Sample characteristics

Non-responders more often reported a history of psychiatric illness. Otherwise, no differences in sociodemographic and clinical characteristics were noted between responders and non-responders (S1 Table).

Sociodemographic and clinical characteristics of the study population by tumor stage are shown in Table 1. There was a difference in the distribution of cancer diagnoses between stage I and advanced cancer; the most common diagnosis in the advanced gynecological cancer group was ovarian cancer (52.7%), whereas endometrial cancer (65.3%) dominated the stage I group. Women with advanced cancer were on average older (mean 64 vs 58 years) and received a greater number of treatments than women in stage I. In addition, they less frequently reported a history of psychiatric illness (7% vs 18%), and were more often living with a partner (76% vs 68%). No significant differences were found in alcohol consumption, smoking, ASA score or BMI.

**Table 1:** Sociodemographic and clinical characteristics of the study population stratified by tumor stage.

	FIGO stage I n = 222 (59.6%)	FIGO stage ≥ II n = 150 (40.3%)	p -value
**Cancer diagnosis**			**0.000**
Ovarian	12 (5.4)	79 (52.7)	
Endometrial	145 (65.3)	63 (42.0)	
Cervical	65 (29.3)	8 (5.3)	
Number of treatment modalities*			**0.000**
1	165 (74.3)	19 (12.7)	
2	51 (23.0)	113 (30.4)	
3	6 (2.7)	18 (4.8)	
**Age, year** (mean, range)	58 (25–86)	64 (24–85)	**0.000**
**BMI, kg/m** ^**2**^	27 (18–61)	26 (19–49)	0.188
**<25**	82 (37.1)	60 (39.7)	0.960
**25–30**	79 (35.7)	54 (36.5)	0.498
**>30**	60 (27.2)	36 (23.8)	0.575
**ASA classification**			0.220
10	73 (36.0)	35 (24.6)	
20	108 (53.2)	94 (66.2)	
30	21 (10.3)	13 (9.2)	
40	1 (0.5)	0	
**Number of comorbidities**			0.291
None	95 (43.3)	58 (42.3)	
1	53 (25.9)	40 (29.2)	
2	30 (14.6)	23 (16.8)	
3	15 (7.3)	10 (7.3)	
≥4	12 (5.8)	6 (4.3)	
**History of psychiatric illness**			**0.000**
Yes	34 (17.5)	9 (7.0)	
No	160 (82.5)	120 (93.0)	
**Smoking**			0.603
Yes	23 (10.7)	23 (15.9)	
No	191 (89.3)	122 (84.1)	
**Level of education**			0.063
University	86 (43.2)	42 (31.6)	
Non-university	113 (56.8)	91 (68.4)	
**Living situation**			**0.001**
With partner	124 (68.8)	87 (76.3)	
Living alone	59 (32.2)	27 (23.7)	
**Alcohol consumption**			0.344
Alcohol units per week (mean, range)	2 (0–12)	2 (0–13)	

Data presented as median, range or n (%). Valid percentages shown. Statistics by independent t-tests. Missing BMI n = 1 missing ASA n = 27, missing number of comorbidities n = 30, missing for history of psychiatric illness n = 49, missing smoking n = 13, missing level of education n = 40, missing living situation 45, missing alcohol consumption n = 38.

.BMI = body mass index, ASA = American Society of Anesthesiologist Physical Status *Surgery, chemotherapy or radiation therapy as possible treatment modalities.

### Quality of life

Scores on each of the eight QoL domains at diagnosis and at the one-year follow-up, by FIGO stage, are shown in Table 2. Women with advanced gynecological cancer had lower scores in physical functioning (mean = 71.6 [sd = 28.9] vs mean = 81.8 [sd = 20.7], p = 0.000) and role functioning/physical (mean = 62.6 [sd = 34.0] vs mean = 77.2 [sd = 28.4], p = 0.001) at the time of diagnosis. At the one-year follow-up, no differences between stage groups were observed in these domains. However, women with advanced cancer scored higher in the mental health domain than did women with stage I (mean = 78.3 [sd = 17.0] vs mean = 73.2 [sd = 21.5], p = 0001).

**Table 2:** Mean scores on the SF-36 at diagnosis and one year after diagnosis, by FIGO stage.

	At diagnosis			One year after diagnosis			Group by time interaction
	FIGO stage I n = 222 mean (SD)	FIGO stage ≥ II n = 150 mean (SD)	p value*	FIGO stage I mean (SD)	FIGO stage ≥ II mean (SD)	p value*	P value**
**Physical Functioning**	81.8 (20.7)	71.6 (28.9)	**0.000**	79.8 (22.0)	75.0 (23.6)	0.243	**0.026**
**Role Functioning/Physical**	77.2 (28.4)	62.6 (33.9)	**0.001**	77.8 (28.2)	69.7 (29.4)	0.139	**0.036**
**Bodily Pain**	76.7 (27.2)	70.3 (28.1)	0.947	76.9 (27.2)	77.1 (24.8)	0.153	**0.037**
**General Health**	66.7 (27.2)	65.8 (21.4)	0.517	65.6 (24.1)	66.8 (23.1)	0.476	0.885
**Vitality**	57.2 (24.5)	56.8 (24.3)	0.843	58.7 (25.6)	62. 7 (22.7)	0.081	**0.041**
**Social Functioning**	75.9 (25.6)	71.5 (28.6)	0.126	79.9 (27.1)	81.7 (24.7)	0.109	0.119
**Role Functioning/Emotional**	75.4 (28.8)	74.6 (28.5)	0.547	80.7 (26.9)	81.2 (24.4)	0.155	0.645
**Mental Health**	62. 7 (21.9)	66.6 (19.3)	0.109	73.2 (21.5)	78.3 (17.0)	**0.001**	0.563

*Means compared with t-test

** Change between groups over time by mixed ANOVA

The group by time interaction showed a significant change over time between women with advanced cancer and women in stage I for four of the domains; physical functioning (F(1,367) = 4.99; p = 0.026), role functioning/physical (F(1,355) = 4.44; p = 0.036), bodily pain (F(1,366) = 1.65; p = 0.037) and vitality (F(1,363) = 0.56;p = 0.041). These interactions were driven by women with advanced cancer who reported improvement at the one-year follow-up, whereas the change over time was smaller in women with stage I cancer (Table 2).

A comparison with the Swedish population of the eight QoL domains at the time of diagnosis and at the one-year follow-up is shown in S2A and S2B Table. At the one-year follow-up the scores for physical functioning, role functioning/physical and bodily pain were comparable or higher than the Swedish population for both women with advanced disease and women with stage I gynecologic cancer. However, vitality scores and social functioning were lower than the population norm in both groups; respectively for stage I (mean = 58.7 [sd = 25.6] vs population mean = 67.2, p = 0.000); (mean = 79.9 [sd = 27.10] vs population mean = 86.9, p<0.001) and for advanced stage (mean = 62.7 [sd = 22.69] vs population mean = 67.2, p = 0.016); (mean = 81.7 [sd = 24.65] vs population mean = 86.9, p = 0.010).

Mental health in stage I was lower at the one-year follow-up than in the Swedish population (mean = 73.2 [sd = 21.5] vs population mean = 78.6, p = 0.000) (S2 Table).

The mean scores on the SF-36 domains for women with FIGO stage >II was further analyzed by gynecologic cancer diagnosis (Table 3). Women with advanced ovarian cancer had lower scores than endometrial cancer patients at diagnosis in three domains; role functioning physical (mean = 53.6 [sd = 35.4] vs mean = 72.3 [sd = 28.8], p = 0.01, bodily pain (mean = 65.6[sd = 29.1] vs mean = 76.3[sd = 24.5], p = 0.002) and social functioning (mean = 64.7[sd = 30.9] vs mean = 79.9[sd = 22.3], p = 0.001). No differences in SF-36 domain scores were noted between women with advanced endometrial and ovarian cancer one year after diagnosis.

**Table 3:** Comparison of the mean scores on the SF-36 for FIGO stage ≥II ovarian and endometrial cancer at diagnosis and one year after diagnosis.

	At diagnosis			One year after diagnosis n = mean (SD)		
	Ovarian cancer n = mean (SD)	Endometrial cancer n = mean (SD)	p value*	Ovarian cancer n = mean (SD)	Endometrial cancer n = mean (SD)	p value*
**Physical Functioning**	68.60 (31.13)	75.31 (25.41)	0.160	74.93 (23.15)	74.84 (24.59)	0.982
**Role Functioning/Physical**	53.63 (35.37)	72.27 (28.82)	**0.010**	66.93 (30.80)	71.19 (28.14)	0.394
**Bodily Pain**	65.56 (29.14)	76.27 (24.50)	**0.020**	76.27 (26.65)	78.33 (22.76)	0.625
**General Health**	63.67 (22.02)	68.51 (19.69)	0.173	66.42 (22.71)	66.42 (23.89)	0.999
**Vitality**	52.24 (24.22)	62.40 (22.75)	0.012	64.24 (22.71)	61.03 (22.15)	0.398
**Social Functioning**	64.74 (30.93)	79.88 (22.29)	**0.001**	79.58 (28.17)	83.01 (20.21)	0.416
**Role Functioning/Emotional**	70.95 (30.73)	77.73 (25.89)	0.173	80.59 (24.77)	80.73 (24.57)	0.400
**Mental Health**	54.62 (19.23)	68.36 (18.98)	0.248	78.73 (16.43)	77.24 (18.10)	0.973

*Means compared with t-test

Sociodemographic and clinical characteristics in relation to the eight QoL domains at the one-year follow-up are shown in Table 4A (for physical health domains) and B (for mental health domains). S3 Table provides this information divided by diagnosis.

**Table 4:** Multiple regression analyses for physical health (A) and mental health (B) one year after diagnosis.

**A**				
	**Physical Functioning**	**Role Functioning/Physical**	**Bodily Pain**	**General Health**
	**B (95% CI)**	**B (95% CI)**	**B (95% CI)**	**B (95% CI)**
**Age**	**-0.4 (-0.6–0.1)**	-0.1 (-0.4–0.2)	0.0 (-0.3–0.3)	-0.1 (-0.3–0.2)
**BMI 25–30 vs <25**	**-7.9 (-14.9–0.9)**	-4.7 (-14.5–5.1)	-0.7 (-9.6–8.1)	-4.8 (-12.5–2.8)
**BMI >30 vs <25**	**-16.2 (-24.3–0.9)**	**-11.7 (-23.1–0.3)**	-7.6 (-17.9–2.7)	**-9.9 (-18.8–1.0)**
**History of psychiatric illness**	-7.1 (-16.1–2.1)	**-14.1 (-27.1–1.3)**	**-17.1 (-28.7–5.5)**	**-16.2 (-26.1–6.2)**
**Lower education (non-university)**	3.1 (-3.1–9.2)	-1.8 (-10.3–6.8)	1.3 (-6.5–9.1)	-0.7 (-7.5–6.0)
**Smoking**	3.9 (-4.8–12.7)	-3.5 (-15.7–8.7)	-0.7 (-11.9–10.5)	-3.6 (-13.3–6.1)
**Number of comorbidities**	-1.7 (-4.4–0.8)	-3.4 (-6.9–0.3)	-3.1 (-6.4–0.2)	-2.9 (-5.8–0.1)
**FIGO stage ≥ II**	4.6 (-3.1–12.4)	2.1 (-8.4–12.8)	4.4 (8–5.3–14.6)	-2.4 (-10.4–5.6)
**Number of treatment modalities >1**	**-7.9 (-15.2–0.8)**	**-10.9 (-21.1–7.1)**	-5.3 (-14.6–4.0)	-2.4 (-10.4–5.6)
**Living alone**	-0.9 (-4.2–2.4)	-1.1 (-5.7–3.4)	0.0 (-4.1–4.2)	-0.1 (3.4–0.9)
**Alcohol consumption**	-0.1 (-1.4–1.1)	-0.5 (-2.2–1.2)	0.6 (-0.9–2.2)	0.2 (-1.1–1.6)
**B**				
	**Vitality**	**Social Functioning**	**Role Functioning/Emotional**	**Mental Health**
	**B (95% CI)**	**B (95% CI)**	**B (95% CI)**	**B (95% CI)**
**Age**	0.2 (-0.1–0.5)	**0.4 (0.1–0.6)**	0.3 (-0.0–0.5)	0.2 (-0.0–0.4)
**BMI 25–30 vs <25**	**-8.9 (-16.8–1.1)**	-5.1 (-13.6–3.4)	-5.4 (-13.7–2.8)	-3.4 (-9.8–3.1)
**BMI >30 vs <25**	-7.8 (-17.0–1.3)	-9.1 (-19.0–0.7)	**-12.2 (-21.8–2.6)**	-6.4 (-13.9–1.1)
**History of psychiatric illness**	**-16.3 (-26.6–6.1)**	-10.8 (-21.9–0.2)	**-14.6 (-25.4–3.6)**	**-12.9 (-21.3–4.6)**
**Lower education (non-university)**	-1.6 (-8.5–5.3)	-5.3 (-12.8–2.1)	1.7 (-5.5–9.0)	-0.5 (-6.1–5.2)
**Smoking**	-0.0 (-9.8–9.9)	-7.4 (-18.1–3.3)	-7.8 (-18.2–2.4)	-0.1 (-8.2–7.9)
**Number of comorbidities**	-1.8 (-4.7–1.1)	-2.9 (-6.1–0.2)	**-3.6 (-6.7–0.6)**	-1.6 (-4.1–0.7)
**FIGO stage≥ II**	5.2 (-3.4–13.8)	23 (-6.9–0.2)	4.5 (-4.5–13.5)	3.7 (-3.2–10.8)
**Number of treatment modalities >1**	-1.6 (-9.8–6.6)	-2.6 (-11.4–6.3)	-5.9 (-14.6–2.7)	-1.2 (-7.9–5.5)
**Living alone**	-1.3 (-5.0–2.4)	-1.6 (-5.6–2.4)	-3.8 (-7.7–0.1)	-1.7 (-4.8–1.3)
**Alcohol consumption**	-0.3 (-1.7–1)	-1.2 (-2.7–0.3)	-0.9 (-2.4–0.5)	-0.5 (-1.6–0.7)

B: Beta unstandardized coefficient, CI: Confidence Interval. Significant values (P<0.05) marked as bold.

Older age, higher BMI and more than one treatment modality were associated with poorer physical functioning (Table 4A). In terms of role functioning, BMI over 30, a history of psychiatric illness and higher number of treatment modalities were related to lower scores. For bodily pain only a history of psychiatric illness was associated with lower scores and a history of psychiatric illness was further associated with general health, where in addition BMI over 30 was associated with a lower score.

For the mental health domains (Table 4B), BMI 25–30 and a history of psychiatric illness were associated with lower scores on vitality. History of psychiatric illness was also related to poorer scores in mental health and the role functioning/emotional domain. Only younger age was associated with lower scores on social functioning, while for role functioning/emotional, a BMI over 30 and comorbidities were associated to lower scores.

Smoking, level of education, FIGO stage, alcohol consumption and living situation were not associated with any of the eight QoL domains.

## Discussion

Interestingly, in this study, women with advanced gynecological cancer reported equally good QoL compared to women with less advanced disease, and they even had better scores on mental health one year after diagnosis. Furthermore, they had greater improvements over time for four health domains than women with local disease. On the other hand, a history of psychiatric illness and higher BMI were consistently associated with poorer QoL at the one-year follow-up on both physical and mental health domains. In addition, more than one treatment modality was associated with lower physical QoL one year after diagnosis.

Previous studies on QoL in gynecological cancer survivors are heterogeneous, including different distribution of FIGO stage and within a different time frame from diagnosis and treatment. Several studies conclude that gynecological cancer survivors have lower QoL compared to healthy individuals [27–29] with higher rates of depression, anxiety and functional impairments. In contrast, there are studies that find a good QoL among gynecological cancer survivors. In a review article by Ahmed-Lecheheb et al. [17], women with ovarian cancer generally had good QoL compared to healthy women despite the persistence of psychological and physical symptoms. In addition, a later study examined QoL in advanced ovarian cancer survivors and found higher scores among these women than among healthy individuals when 8.5 or more years had passed since diagnosis, while their levels of physical activity were lower [18]. Furthermore, in a prospective study of Greimel et al. [4] estimating QoL of gynecological cancer patients at six points pre-to post treatment, their QoL was found comparable to breast cancer patients at one year follow-up, suggesting a significant improvement in QoL following treatment. This is supported by another prospective study finding most improvements in QoL at six months from treatment [12]. This suggests that gynecological cancer patients adjust over time to their physical changes in recovery.

In our study we compared advanced gynecological cancer survivors to those in tumor stage I in order to elucidate the impact of higher tumor load, worse prognosis and more compelling treatment (often with more than one treatment modality) on QoL. Furthermore, advanced ovarian cancer is nowadays treated with a more aggressive surgical approach [30], hence results from older studies might not be valid today. We hypothesized that extensive surgery with potential long-time consequences would lead to worse QoL; however, this was not confirmed in our study population. The difference in physical and role functioning was only noted at diagnosis, supporting the findings of previous studies that most improvements in QoL occur within the first year [3]. However, we did find that more than one treatment modality was associated with lower scores in the physical functioning and role functioning physical domains one year after diagnosis suggesting that more extensive treatment can lead to physical sequela. Further, we did not find any difference in the QoL scores for women with advanced cancer one year after diagnoses when stratifying by histology.

In our study the main predictive factors for both mental and physical health domains were a history of psychiatric illness, and higher BMI. Regarding psychiatric illness it has previously been shown that women with gynecological cancer have a higher prevalence of clinical depression than healthy individuals at diagnosis. Furthermore, it is suggested that within five years of diagnosis the prevalence becomes equal to healthy controls [5], however further studies are needed in this regard. In addition, it is not known how the psychiatric history affects quality of life, but in our study, it was a predictor of lower mental and physical QoL. Surprisingly, women with stage I disease had lower scores on mental health than women with advanced cancer one year after diagnosis, which might be due to a slightly higher rate of a history of psychiatric illness in this group. Future studies should examine in detail which psychiatric diagnoses are predictive of worse outcome, and special attention should be given to women with a history of psychiatric illness at diagnosis.

BMI has previously been identified as a risk factor for poor QoL, further supported by a recent review on QoL in endometrial cancer [19]. Presumably, this is due to higher BMI being associated with lower activity levels and physical functioning [31]. Furthermore, similar findings have been seen in otherwise healthy individuals (without cancer) [32] and in gynecological cancer patients before treatment, compared with patients with normal BMI [33]. Accordingly, special attention should be paid to obesity in the follow-up of gynecologic cancer patients. Furthermore, the QoL outcomes in our study were compared to the normal Swedish population and in regard to physical domains the study population did not have worse outcomes. However, regarding vitality and social functioning they had lower scores.

The strengths of this study are its relatively large size, with patients treated at the same hospital using a validated questionnaire to assess QoL at diagnosis and after one year. The prospective approach minimizes the risk of recall bias. The self-reported information on comorbidity and psychiatric illness was completed with information from medical records, which increases the validity of the data. We also had the advantage of including potential sociodemographic confounders such as smoking, alcohol habits, level of education and living situation. The study included three gynecological cancer types, which could be seen as a limitation; however, we hypothesized that tumor load and the number of treatment modalities reflected in a higher tumor stage were more important factors than the actual tumor site, which was suggested in one previous study [34].

Some limitations should be addressed. Thirty-two percent of women answering the questionnaire at diagnosis did not return the one-year questionnaire. These women more often had a history of psychiatric illness. This might indicate that our study cohort had a higher level of well-being than the entire gynecological cancer population. Additionally, information concerning psychiatric illness was reported at the time of the cancer diagnosis; consequently the answers might be influenced by the situation. Further, the questionnaire SF-36 is not specifically designed to estimate the quality of life of cancer patients.

Our results indicate an overall good QoL one year after diagnosis of gynecological cancer. In spite of this, there are women that health care providers should give special attention to at diagnosis without regard to tumor stage: women with a history of psychiatric illness and obesity. The development of minimal invasive surgery and ERAS (enhanced recovery after surgery) protocols is positive in many aspects, but there is a risk that with a shorter hospital stay, the need for physical and psychological rehabilitation might be overlooked. This is especially important for women with psychiatric illness, who might not have the strength to seek help once they are discharged. Perhaps developing a risk score for women with planned gynecologic cancer treatment is possible to identify the women in need of additional psychiatric support, in line with comorbidity scores already introduced in order to identify women with worse oncologic outcomes [35]. There have already been studies showing that psychosocial interventions can improve QoL [36] and further studies should focus on the implementation of such methods.

## Conclusion

The QoL of women with advanced gynecological cancer appears to improve over time with regards to physical functioning, role functioning physical, bodily pain and vitality. One year after diagnosis these women report better QoL compared to women with limited disease and even higher scores on mental health one year after diagnosis. However, women with previous psychiatric illness and high BMI reported more impaired physical and mental health and may need additional support. Greater attention to women with these risk factors may be warranted before the start of treatment.

## Supporting information

S1 Table Attrition analysis between responders and non-responders. (DOCX)

S2 Table **(A)**: Mean score on the SF36 at diagnosis by FIGO stage and the Swedish population, females in age group 60–64. **(B)**: Mean scores on the SF36 one year after diagnosis in the study population by FIGO stage, and in the general Swedish population.
(DOCX)

S3 Table Multiple regression analyses for physical health (A) and mental health (B) after one year by diagnosis.
(DOCX)
