# Supplementary material for: Advanced gynecological cancer: Quality of life one year after diagnosis
Source: PLoS One. 2023 Jun 23;18(6):e0287562. doi: 10.1371/journal.pone.0287562 (PMC10289468; doi:10.1371/journal.pone.0287562)
Supplement: S1 Table — (DOCX) [file pone.0287562.s001.docx]

|  | **Responders**  n=372 | **Non-responders**  n=177 |  | **p value** |
| --- | --- | --- | --- | --- |
| **Cancer diagnosis** |  |  |  | 0.076 |
| Ovarian | 91 (24.5) | 59 (33.3) |  |  |
| Endometrial | 208 (56.5) | 86 (48.6) |  |  |
| Cervical | 73 (19.1) | 32 (18.1) |  |  |
| **Age, year** (median, range) | 64 (24-86) | 61(21-88) |  | 0.640 |
| **BMI, kg/m^2^** | 26.7 (17-61) | 26.0 (16-50) |  | 0.355 |
| **ASA classification** |  |  |  | 0.477 |
| 10 | 100 (31.1) | 44 (27.2) |  |  |
| 20 | 202 (58.4) | 99 (61.1) |  |  |
| 30 | 34 (10.2) | 19 (11.7) |  |  |
| 40 | 1 | 0 |  |  |
| **FIGO stage** |  |  |  | 0.06 |
| I | 222 (59.6) | 99 (55.9) |  |  |
| ≥ II | 150 (40.4) | 78 (44.1) |  |  |
| **History of psychiatric illness** |  |  |  | **0.001** |
| Yes | 43 (12.7) | 23 (18.9) |  |  |
| No | 282 (87.3) | 99 (81.1) |  |  |
| **Smoking** |  |  |  | 0.505 |
| Yes | 46 (12.8) | 15 (9.1) |  |  |
| No | 312 (87.2) | 150 (90.9) |  |  |
| **Level of education** |  |  |  | 0.984 |
| University | 128 (38.1) | 51 (42.1) |  |  |
| Non-university | 204 (61.9) | 70 (57.9) |  |  |
| **Living situation** |  |  |  | 0.384 |
| With partner | 211(70.7) | 89 (72.9) |  |  |
| Living alone | 86 (29.3) | 33 (27.1) |  |  |
| **Alcohol consumption** |  |  |  | 0.107 |
| Alcohol units per week (median, range) | 1 (0-13) | 2 (0-10) |  |  |

**Supplementary Figure 1:** **Attrition analysis between responders and non-responders.**

Percentages presented in relation to available information. Valid percentages shown. Statistics by independent T-tests. Missing BMI n=6 missing ASA n=42, Missing for history of psychiatric illness n=104, missing smoking n=87, missing level of education n=96, missing living situation 122, missing alcohol consumption n= 98.

BMI=body mass index, ASA=American Society of Anesthesiologist Physical Status
