# Supplementary material for: Advanced gynecological cancer: Quality of life one year after diagnosis
Source: PLoS One. 2023 Jun 23;18(6):e0287562. doi: 10.1371/journal.pone.0287562 (PMC10289468; doi:10.1371/journal.pone.0287562)
Supplement: S2 Table — (A): Mean score on the SF36 at diagnosis by FIGO stage and the Swedish population, females in age group 60–64. (B): Mean scores on the SF36 one year after diagnosis in the study population by FIGO stage, and in the general Swedish population. (DOCX) [file pone.0287562.s002.docx]

**Supplementary figure 2A:** **Mean score on the SF36 at diagnosis by FIGO stage and the Swedish population, females in age group 60-64.**

|  | **All**  n=372  mean (SD) | **FIGO stage I**  n=222  mean (SD) | **FIGO stage ≥ II**  n=150  mean (SD) | **p value** | **Swedish female population** (age 60-64)  mean | **p value**  **FIGO stage I** | **p value FIGO stage≥ II** |
| --- | --- | --- | --- | --- | --- | --- | --- |
| **Physical Functioning** | 77.7 (24.8) | 81.8 (20.7) | 71.6 (28.9) | **0.000** | 76.2 | **0.000** | 0.053 |
| **Role Functioning/Physical** | 71.4 (31.5) | 77.2 (28.4) | 62.6 (34.0) | **0.001** | 70.5 | **0.001** | **0.006** |
| **Bodily Pain** | 74.1 (27.7) | 76.7 (27.2) | 70.3 (28.1) | 0.947 | 64.8 | **0.000** | **0.019** |
| **General Health** | 66.3 (22.9) | 66.7 (27.2) | 65.8 (21.4) | 0.517 | 66.2 | 0.742 | 0.804 |
| **Vitality** | 57.3 (22.9) | 57.2 (24.5) | 56.8 (24.3) | 0.843 | 67.2 | **0.000** | **0.000** |
| **Social Functioning** | 73.6 (26.9) | 75.9 (25.6) | 71.5 (28.6) | 0.126 | 86.9 | **0.000** | **0.000** |
| **Role Functioning/Emotional** | 75.1 (28.6) | 75.4 (28.8) | 74.6 (28.5) | 0.547 | 80.1 | **0.017** | **0.024** |
| **Mental Health** | 64.3 (20.9) | 62. 7 (21.9) | 66.6 (19.3) | 0.109 | 78.6 | **0.000** | **0.000** |

**Supplementary Figure 2B:** **Mean scores on the SF36 one year after diagnosis in the study population by FIGO stage, and in the general Swedish population.**

|  | **All**  n=372  mean (SD) | **FIGO stage I**  mean (SD) | **FIGO stage ≥ II**  mean (SD) | **p value** | **Swedish female population** (age 60-64)  mean | **p value**  **FIGO stage I** | **p value FIGO stage≥ II** |
| --- | --- | --- | --- | --- | --- | --- | --- |
| **Physical Functioning** | 77.8 (22.7) | 79.8 (22.0) | 75.0 (23.6) | 0.243 | 76.2 | **0.017** | 0.536 |
| **Role Functioning/Physical** | 74.5 (28.9) | 77.8 (28.2) | 69.7 (29.4) | 0.139 | 70.5 | **0.000** | 0.729 |
| **Bodily Pain** | 77.0 (26.3) | 76.9 (27.2) | 77.1 (24.8) | 0.153 | 64.8 | **0.000** | **0.000** |
| **General Health** | 66.1 (23.7) | 65.6 (24.1) | 66.8 (23.1) | 0.476 | 66.2 | 0.709 | 0.737 |
| **Vitality** | 60.3 (24.5) | 58.7 (25.6) | 62. 7 (22.7) | 0.081 | 67.2 | **0.000** | **0.016** |
| **Social Functioning** | 80.6 (26.1) | 79.9 (27.1) | 81.7 (24.7) | 0.109 | 86.9 | **0.000** | **0.010** |
| **Role Functioning/Emotional** | 80.9 (25.9) | 80.7 (26.9) | 81.2 (24.4) | 0.155 | 80.1 | 0.749 | 0.593 |
| **Mental Health** | 72.3 (19.9) | 73.2 (21.5) | 78.3 (17.0) | **0.001** | 78.6 | **0.000** | 0.848 |
