# Supplementary material for: Advanced gynecological cancer: Quality of life one year after diagnosis
Source: PLoS One. 2023 Jun 23;18(6):e0287562. doi: 10.1371/journal.pone.0287562 (PMC10289468; doi:10.1371/journal.pone.0287562)
Supplement: S3 Table — Multiple regression analyses for physical health (A) and mental health (B) after one year by diagnosis. (DOCX) [file pone.0287562.s003.docx]

**Supplementary Figure 3A:** Multiple regression analyses for physical health (A) and mental health (B) after one year by diagnosis.

**A**

|  | **Physical Functioning**  **B (95% CI)** | **Role Functioning/Physical**  **B (95% CI)** | **Bodily Pain**  **B (95% CI)** | **General Health**  **B (95% CI)** |
| --- | --- | --- | --- | --- |
| **Age** | **-3.7 (-0.6—0.4)** | -0.1 (-0.4-0.2) | 0.0 (-0.3-0.3) | -0.1 (0.7—30.3) |
| Endometrial cancer | **-1.0 (-1.5—0.6)** | -0.6 (-1.2—0.0) | -0.5 (-1.1-0.0) | -0.3 (-0.7-0.8) |
| Cervixcancer | -0.1 (-0.7-0.4) | -0.1 (-1.2-0.8) | 0.2 (-0.9-1.3) | 0.2 (-0.4-0.9) |
| Ovarian cancer | -0.3 (-1.1-0.5) | -1.1 (-2.6-0.4) | -0.5 (-1.7 -0.8) | -0.4 (-1.6-0.7) |
| **BMI** | **-1.2 (-1.8—0.7)** | **-1.1 (-1.9—0.4)** | **-0.7 (-1.4—0.0)** | **-0.8 (-1.4—0.3)** |
| Endometral cancer | **-1.4 (-2.1—0.7)** | **-1.4 (-2.3—0.5)** | **-0.9 (-1.8—0.1)** | -0.7 (-1.5-0.5) |
| Cervixcancer | -0.6 (-2.1-0.8) | -0.4 (-2.9-2.1) | -0.0 (-37.6-9.0) | -0.2 (-1.9-1.5) |
| Ovarian cancer | -1.5 (-3.3-0.2) | -3.0 (-6.2-0.1) | -1.2 (-3.9-1.4) | **-1.3 (-3.6-1.1)** |
| **History of psychiatric illness** | -7.5 (-16.3-1.4) | **-13.9 (-26.5—1-4)** | **-17.2 (-28.6—5.9)** | **-16.2 (-25.9—6.4)** |
| Endometrial cancer | -8.7 (-21.7-4.2) | -11.9 (-28.8-5.0) | **-19.2 (-4.1-0.8)** | **-18.9 (-33.2—4.6)** |
| Cervixcancer | -5.5 (-18.6-7.6) | -13.8 (-36.2-8.6) | -14.3 (-37.6-9.0) | **-16.1 (-31.7—0.5)** |
| Ovarian cancer | **-78.8 (-132.2—25.4)** | -70.4 (-166.4-25.5) | -57.9 (-138.7-22.8) | -32.7 (-109.7-38.2) |
| **Lower education (non-university)** | 2.5 (-3.6-8.6) | -2.8 (-11.3-5-6) | 0.5 (-7.3-8.2) | -1.3 (-8.1-5.3) |
| Endometrial cancer | 2.2 (-6.3 -10.7) | 0.4 (-10.5-11.3) | -0.2 (-10.5-9.7) | -1.4 (-10.9-7.9) |
| Cervixcancer | -0.2 (-12.1-11.7) | -9.1 (-29.0-10.8) | -2.9 (-23.6 -17.8) | -5.8 (-19.7-8.1) |
| Ovarian cancer | -0.4 (-21-2-19-4) | -19.0 (-54.6-16.5) | 0.5 (-29.5-30.4) | 3.2 (-23.1-29.5) |
| **Smoking** | 3.9 (-4.7-12.5) | -3.5 (-15.4-8.5) | -0.4 (-11.4-10.7) | -3.6 (-13.2-5.8) |
| Endometrial cancer | 8.1 (-4.18-20.5) | -4.6 (-20.4-2.5) | 3.8 (-10.5-18.3) | -3.9 (-17.6-9.7) |
| Cervixcancer | -13.5 (-31.9-4.8) | -3.4 (-23.3-16.5) | -20.9 (-53.6-11.7) | -19.4 (-41.4-2.5) |
| Ovarian cancer | 24 (-18.0-22.7) | -5.7 (-42.3-30.9) | -0.4 (-31.2-30.4) | 0.3 (-26.7-27.4) |
| **Number of comorbidities** | -1.4 (-3.9-1.2) | -2.7 (-6.3-0.8) | -2.7 (-6.1-0.5) | -2.6 (-5.4-0.2) |
| Endometrial cancer | -0.7 (-3.7-2.4) | -3.1 (-7.1-0.7) | -2.1 (-5.8-1.4) | -2.9 (-6.3-0.5) |
| Cervixcancer | -9.8 (-23.6 -4.0) | 3.4 (-23.3—16.5) | -9.5 (-30.2-11.8) | -9.6 (-23.6-4.2) |
| Ovarian cancer | -4.7 (-12.7-3.3) | 3-4 )-10.9-17.9) | -3.8 (-15.9-8.3) | -2.2 (-12.8-8.5) |
| **FIGO stage ≥ II** | 4.9 (-2.5-12.5) | 2.4 (-7.9-12.8) | 4.1 (-5.4-13.7) | 3.0 (-5.1-11.2) |
| Endometrial cancer | 4.1 (-6.1-14.3) | 3-0 (-9.7-15.8) | 2.4 (-9.3-14.2) | 2.5 (-8.6-13.6) |
| Cervixcancer | 18.6 (-2.9-40.2) | 35.4 (0.7-70.0 | 30.3 (-5.7-66.3) | **29.1 (4.8-53.2)** |
| Ovarian cancer | 0.6 (-23.5-24.7) | -7.3 (-50.7-36.1) | 3.1 (-33.4-39.6) | 7.6 (-24.4-39.7) |
| **Number of treatment modalities >1** | **-9.2 (-16.5—2.0)** | **-12.3 (-22.4--2.3)** | -5.8 (-15.1-3.4) | -3.4 (-11.4-4.5) |
| Endometrial cancer | -0.7 (-16.0-1.9) | -8.9 (-20.4-2.4) | -2.6 (-13.0-7.8) | 1.2 (-8.7-11.0) |
| Cervixcancer | **-19.3 (-33.2—5.4)** | -22.3 (-46.2-1.5) | -17.2 (-42.1-7.6) | -10.9 (-27.6-5.7) |
| Ovarian cancer | -45.6 (-94.6-3.4) | -48 (-136.2-40-1) | -48.9 (-123.2-25.2) | -63.6 (-128.7-1.6) |
| **Living alone** | -0.6 (-3.9-2.5) | -0.8 (-5.4-3.7) | 0.2 (-3.9-4.3) | 0.0 (-3.5-3.6) |
| Endometrial cancer | 0.7 (-3.6-5.2) | -0.1 (-5.7-5.4) | 0.3 (-4.8-5.4) | 1.1 (-3.7-5.8) |
| Cervixcancer | -0.7 (-7.5-6.1) | 3.7 (-7.9-15.3) | 4.8 (-7.4-17.1) | -0.2 (-8.3-8.0) |
| Ovarian cancer | -6.0 (-13.8-1.8) | -7.5 (-21.7-6.6) | -6.4 (-18.4-5.5) | -6.8 (-17.3-3.6) |
| **Alcohol consumption** | -0.9 (-1.4-1.0) | -0.59 (-2.3-1.1) | 0.6 (-0.9-2.2) | 0.2 (-1.1-1-5) |
| Endometrial cancer | 0.1 (-1.6-1.8) | -0.6 (-2.7 -1.6) | 0.8 (-1.1-2.8) | 0.7 (-1.2-2.9) |
| Cervixcancer | 0.1 (-2.9-3.1) | 0.0 (-5.1-5.2) | 1.1 (-4.1-6.5) | 1.2 (-2.4-4.6) |
| Ovarian cancer | 1.4 (-1.9-4.7) | 1.4 (-4.5-7.3) | 1.2 (-3.7-6.2) | -0.6 (-4.9-3.7) |

B: Beta unstandardized coefficient, CI: Confidence Interval. Significant values (P<0.05) marked as bold.

**B**

|  | **Vitality**  **B (95% CI)** | **Social Functioning**  **B (95% CI)** | **Role Functioning/Emotional**  **B (95% CI)** | **Mental Health**  **B (95% CI)** |
| --- | --- | --- | --- | --- |
| **Age** | 0.2 (-0.1-0.4) | **0.3 (0.1-0.6)** | 0.3 (-0.0-0.5) | 0.2 (0.1—0.0) |
| Endometrial cancer | **-0.1(-0.5-0.4)** | 0.2 (-0.3-0.7) | 0.0 (-0.4-0.5) | 0.2 (-0.3-0.6) |
| Cervixcancer | 0.2 (-0.8-1.2) | 0.1 (-0.8—0.8) | 0.4 (-5.1-1.3) | 0.3 (-0.3-0.9) |
| Ovarian cancer | -0.8 (-1.9-0.2) | -0.7 (-2.1-0.6) | -0.3 (-1.4-0.8) | -0.3 (-1.1-0.5) |
| **BMI** | **-0.7 (-1.4—0.1)** | **-0.9 (-1.5—2.3)** | **-1.1 (-1.7—0.4)** | **-0.6 (-1.1—0.1)** |
| Endometral cancer | -0.7 (-1.4-0.0) | -0.7 (-1.5-0.0) | **-0.9 (-1.7—0.9)** | -0.5 (-1.2-0.1) |
| Cervixcancer | -0.7 (-3.2-1.7) | -1.3 (-3.5-0.9) | **-2.7 (-4.9—0.5)** | -0.7 (-2.3-0.8) |
| Ovarian cancer | **-2.5 (-4.8-10.3)** | -2.6 (-5.4-0.2) | -1.4 (-3.7-0.8) | -0.9 (-2.3-0.8) |
| **History of psychiatric illness** | **-15,9 (-25.9—5.7)** | -10.6 (-21.4-0.2) | **-14.7 (-25.3-4.0)** | **-12.8 (-21.0—4.6)** |
| Endometrial cancer | -8.7 (-21.7-4.3) | -2.7 (-16.7-11.4) | -14.6 (-29.7 -0.4) | -5.8 (-18.3-6.5) |
| Cervixcancer | -18.0 (-40.4-4.3) | **-16.1 (-36.1-3.9)** | -3.0 (-23.1-17.0) | **-17.6 (-31.7—3.6)** |
| Ovarian cancer | -42.0 (-109.0-24.9) | -39.6 (-124.7-45.6) | **-86.7 (-155.8 –17.8)** | -39.1 (-91.3-13.1) |
| **Lower education (non-university)** | -1.8 (-8.7-5.1) | -6.1 (13.5 (1.3) | 0.9 (-6.2-8-1) | -0.9 (-6.6-4.6) |
| Endometrial cancer | -0.2 (-8.8-8.4) | -6.3 (-15.7-2.9) | 0.8 (-8.9-10.4) | -1.4 (-9.6-6.8) |
| Cervixcancer | -7.8 (-27.2-12.1) | -3.6 (-21.4-14.1) | -0.7 (-18.6-17.1) | 1.4 (-11.1) |
| Ovarian cancer | -9.6 (-34.4-15.2) | -13.1 (-44.7-18.4) | -10.7 (-36.3-14.8) | -8.1 (-27.5-11.2) |
| **Smoking** | -0.8 (-10.6-9.1) | -7.6 (-18.1-2.4) | -7.9 (-18.0-2.3) | -0.2 (-8.1-7.6) |
| Endometrial cancer | 0.6 (-11.8-12.9) | -6.6 (-20.0-6.9) | -8.7 (-22.6-5.1) | -1.6 (-13.4-10.3) |
| Cervixcancer | -5.4 (-36.8-25.9) | -0.3 (-28.4-27.8) | -7.7 (-35.8-20.4) | -4.8 (-24.7-14.8) |
| Ovarian cancer | -11.9 (-37.5-13.6) | -20.5 (-53.0-12.0) | -8.4 (-34.7-17.8) | 2.2 (-17.7-22.2) |
| **Number of comorbidities** | -1.4 (-4.4-1.5) | -2.5 (-5.6-0.7) | **-3.1 (-6.2—0.1)** | **-**1.3 (-3.7-1.1) |
| Endometrial cancer | -1.2 (-4.3-1.9) | **-3.4 (-6.7—0.3)** | **-3.8 (-7.3—0.2)** | -1.7 (-4.7-1.3) |
| Cervixcancer | -2.5 (-22.4-17.4) | 6.7 (-11.1-24.5) | 10.9 (-6.9-28.7) | -1.1 (-21.9-8.1) |
| Ovarian cancer | 1.6 (-8.4-11.7) | 1.2 (-11.6-14.0) | -0.6 (-11.0-9.7) | 1.8 (-5.9-9.7) |
| **FIGO stage ≥ II** | 6.5 (-1.9-14.9) | 2.8 (-6.2-11.9) | 4.7 (-4.0-12.5) | 4.1 (-2.8-10.9) |
| Endometrial cancer | 3.5 (-6.5-13.6) | 1.1 (-9.8-12.1) | 4.5 (-6.7-15.9) | 1.7 (-7.9-11.4) |
| Cervixcancer | 22.6 (-11.9-57.2) | **36.4 (5.4-67.3)** | 18.5 (-12.5-49.5) | 18.1 (-3.6-39.9) |
| Ovarian cancer | -4.2 (-34.7-25.9) | -5.5 (-44.1-32.9) | -5.3 (-36.5-25.8) | 2.4 (-21.2-26.1) |
| **Number of treatment modalities >1** | -3.3 (-11.5.4.9) | -3.8 (-12.6-4.9) | -7.2 (-15.8-1.2) | -2.1 (-8.7-4.6) |
| Endometrial cancer | -2.4 (-11-6.8) | 2.5 (-7.2-2.2) | -5.5 (-15.6-4.6) | -0.2 (-8.8-8.4) |
| Cervixcancer | -11.9 (-35.8 -11.9) | **-27.3 (-48.7---6.1)** | -10.9 (-32.3-10.4) | -6.8 (21.9-8.2) |
| Ovarian cancer | -48.2 (-109.7-13.3) | -29.6 (-107.9-48.6) | -23.4 (-86.7-39.9) | -32.4 (-80.4-15.5) |
| **Living alone** | -1.1 (-4.8-2.6) | -1.4 (-5.4-2.5) | -3.6 (-7.4-0.3) | -1.5 (1.4-0.9) |
| Endometrial cancer | -2.6 (-7.0-1.8) | -2.6 (-7.4 -2.2) | -3.8 (-8.8-1.2) | -2.3 (-6.5-1.9) |
| Cervixcancer | 1.9 (-9.8-13.6) | 1.8 (-8.6-12.3) | -1.6 (-12.1-8.9) | 0.2 (-0.7-7.5) |
| Ovarian cancer | -5.0 (-14.9-4.8) | -4.2 (-16.8-8.3) | -4.0 (-14.2-6.2) | -5.8 (-13.6-1.8) |
| **Alcohol consumption** | -0.4 (-4.8-2.6) | -1.3 (-2.8-0.2) | -1.0 (-2.5-0.4) | -0.5 (-0.6-2.3) |
| Endometrial cancer | -0.8 (-2.5-0.9) | -1.3 (-3.1-0.6) | -0.8 (-2.7-1.1) | -0.7 (-2.4-0.9) |
| Cervixcancer | 1.1 (-3.9-6.2) | -0.3 (-4.8-4.3) | -0.7 (-5.3-3.8) | -0.1 (-3.3-3.1) |
| Ovarian cancer | 0.7 (-3.5-4.8) | -0.8 (-6.1-4.4) | 0.5 (4.8-0.5) | -0.4 (-3.6-2.7) |
